# Investigation of Wafer-Level Fabricated Permanent Micromagnets for MEMS

**DOI:** 10.3390/mi13050742

**Published:** 2022-05-07

**Authors:** Mani Teja Bodduluri, Björn Gojdka, Niklas Wolff, Lorenz Kienle, Thomas Lisec, Fabian Lofink

**Affiliations:** 1 Fraunhofer Institute for Silicon Technology (ISIT), Fraunhoferstrasse 1, 25524 Itzehoe, Germany; bjoern.gojdka@isit.fraunhofer.de (B.G.); thomas.lisec@isit.fraunhofer.de (T.L.); fabian.lofink@isit.fraunhofer.de (F.L.); 2 Department of Materials Science, Kiel University, Kaiserstrasse 2, 24143 Kiel, Germany; niwo@tf.uni-kiel.de (N.W.); lk@tf.uni-kiel.de (L.K.)

**Keywords:** integrated rare-earth micromagnets, magnetic MEMS, NdFeB, atomic layer deposition, PowderMEMS

## Abstract

Monolithic integration of permanent micromagnets into MEMS structures offers many advantages in magnetic MEMS applications. A novel technique called PowderMEMS, based on the agglomeration of micron-sized powders by atomic layer deposition (ALD), has been used to fabricate permanent micromagnets on 8-inch wafers. In this paper, we report the fabrication and magnetic characterization of PowderMEMS micromagnets prepared from two different NdFeB powder particle sizes. A remanence of 423 mT and intrinsic coercivity of 924 mT is achieved at the low ALD process temperature of 75 °C, making this process compatible with MEMS technology. The magnetic reversible mechanism in the micromagnets is discussed with the help of the Wohlfarth equation. To ensure the operability of such integrated micromagnets in different application environments, we conducted a set of experiments to systematically investigate the thermal and corrosive stability. NdFeB micromagnets with larger powder particle size (d50 = 25 µm) exhibit high thermal stability in air. Furthermore, the corrosion stability of the micromagnets is significantly improved by an additional silicon oxide passivation layer deposited by plasma-enhanced chemical vapor deposition (PECVD). The presented results demonstrate the durability of PowderMEMS micromagnets, enabling their application in various fields, e.g., microfluidics, sensors, actuators, and microelectronics.

## 1. Introduction

The rapidly increasing demand for inexpensive and reliable microelectronics and the ongoing trend of miniaturization are key drivers for growing interest in the fabrication and integration of microelectromechanical systems (MEMS) into devices. Numerous functions of today’s MEMS are based on the integration of magnetic structures. In general, magnetic interaction between permanent magnets benefits from downscaling [1]. For instance, in the case of interaction between two permanent magnets, a decrease of the magnetic system’s dimensions by a scale factor S results in a relative increase in force per unit volume by S^1^ [1]. This is because the field gradient increases by a factor of S^1^ upon miniaturization, as the field strength around the scaled magnet is maintained. Accordingly, MEMS devices with integrated magnetic materials are potential candidates for applications in a wide range of fields, such as energy harvesting, sensors, telecommunication, and life science among others [2,3,4,5,6]. 

Controllable magnetic fields can be produced by micro-electromagnets by adjusting the electric current flow in the coils surrounding the magnetic core. However, on-chip joule heating and an increasing complexity of coil fabrication limit the fields of application [3]. Alternatively, permanent magnetic materials have become preferential passive components employed in the fabrication of magnetic MEMS. Once magnetized, permanent magnets generate continuous forces and torques for actuation at longer distances unlike electrostatic and thermal mechanisms [7]. An application example of magnetic non-contact actuation is provided by energy-harvesting devices where electrical energy is generated by exciting the cantilever having magnet as tip mass [8]. Permanent magnets prepared from rare earth transition metal (RE-TM) alloy materials are the dominant materials due to their high coercive fields and thus a superior robustness and stability against environmental interferences [9].

To efficiently integrate a RE-TM permanent magnet into MEMS platforms, the process technology should enable the fabrication of voluminous, three-dimensional magnetic microstructures at wafer-level. For instance, rare earth permanent micromagnets of thicknesses less than 160 µm and properties similar to bulk sintered magnets can be fabricated using conventional bottom-up physical vapor deposition techniques, such as sputtering and pulsed-laser deposition (PLD) [10,11]. However, these processes are hindered due to large residual stress in the films and require elevated post-annealing temperatures to crystallize the films for realizing good magnetic properties. The annealing can impair prefabricated components and photoresist layers. Besides structural thickness, precise geometries of different dimensions and shapes are required for most micromagnetic applications. Alternatively, thicker and more complex geometries can be fabricated as bonded magnets using low-cost established top-down processes. Bonded micromagnets are fabricated using binding agents, e.g., polymers (PDMS, SU8, Parylene, etc.) and wax [12,13,14]. However, the usage of organic materials is limited by their low glass transition temperatures resulting in poor thermal stability at elevated temperatures. For instance, wax bonded magnets are limited by a comparatively low melting temperature (approximately 180 °C) of wax [12].

**Figure 1:**
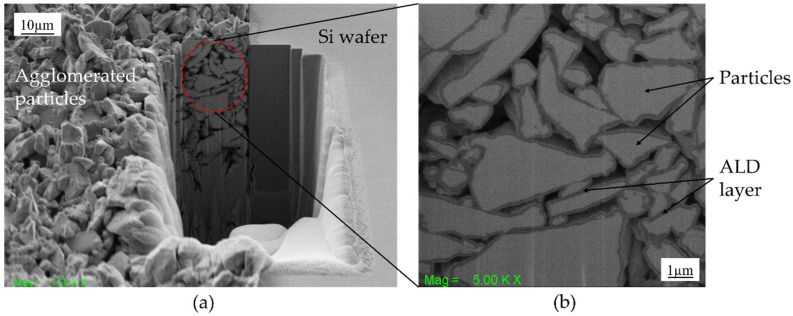
(**a**,**b**) FIB-SEM cross-sectional image of agglomerated particles after atomic layer deposition of aluminum oxide layer.

A novel technique called PowderMEMS is utilized to fabricate high-performance, organic-free RE-TM micromagnets, which are well suited for magnetic MEMS [15]. Based on the agglomeration of powder materials by atomic layer deposition (ALD), the technique is suited for the fabrication of mechanically stable micromagnets of different magnetic materials [15,16]. The fabricated micromagnets have the advantage of being protected against humid environments as the magnetic particles are fully covered by a pinhole free ALD layer. As an example, Figure 1a shows a FIB cross-section SEM image of a NdFeB micromagnet fabricated with the PowderMEMS technique. The SEM image at higher magnification in Figure 1b illustrates that the individual particles of the structure are fixated to each other by an ALD layer. PowderMEMS enables the transfer of macroscopic magnetic solutions into MEMS devices at wafer-level by overcoming the constraints experienced by micromagnets fabricated using conventional MEMS fabrication techniques. Since all steps of the PowderMEMS process can be conducted at wafer-level, and in the case of the ALD deposition even in batches, the PowderMEMS technique can be scaled for mass production. A corresponding line for 200 mm wafers was already demonstrated [15].

In this article, the fabrication, magnetic properties, nanostructure, and durability of ALD agglomerated RE-TM micromagnets are studied. To assess the ALD penetration behavior into porous structures in dependency of process temperature, NdFeB powders of two different particle sizes are investigated. Additionally, the micromagnets are subjected to heat treatments ex situ and in situ in a transmission electron microscope (TEM) to investigate the irreversible and reversible losses. Results of water immersion tests and accelerated aging tests are presented to assess the suitability of the magnets in MEMS applications. 

## 2. Experimental

### 2.1. Micromagnet Fabrication

Dry NdFeB powders from Magnequench with similar composition (including 5–8 at% of Co) and irregular morphology (MQFP−B+10215) were used, featuring two different particle size distributions (d50 = 5 µm and 25 µm). The remanence $B_{r}$, intrinsic coercivity $H_{\mathrm{ic}},$ and maximum energy product density ${\mathrm{BH}}_{\max}$ of the NdFeB powders are 865–895 mT, 701–836 kA/m, and 114–126 $\mathrm{kJ}/m^{3}$, respectively [17]. In the following, the magnetic structures filled with 5 µm and 25 µm NdFeB powders are entitled as NdFeB5 and NdFeB25, respectively.

**Figure 2:**
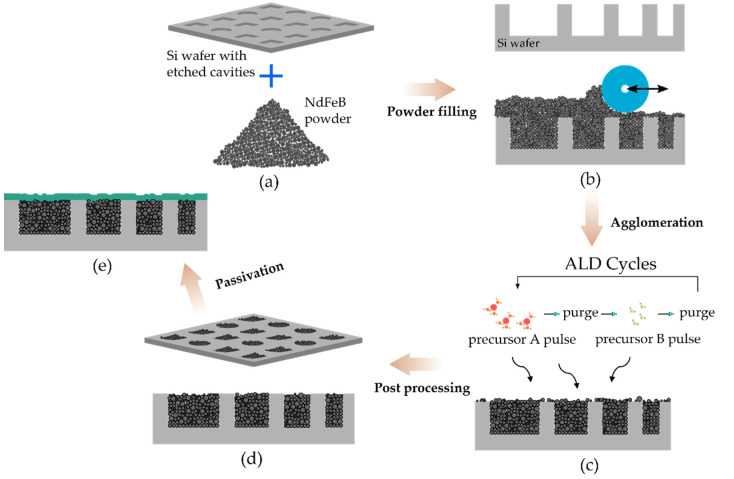
Schematic image depicting the process flow for fabricating ALD-solidified NdFeB micromagnets. (**a**) Etching of cavities of different shapes and sizes into a silicon wafer using DRIE. (**b**) Filling of dry NdFeB powder into pre-etched cavities. (**c**) Deposition of ALD layer to fixate the loose particles. (**d**) Post-processing of substrate wafer. (**e**) Passivation of the fabricated structures with SiO_2_ layer deposited by PECVD.

Cavities of different shapes and sizes were etched into an 8-inch silicon wafer (Figure 2a) by deep reactive ion etching (DRIE), with depths varying from 100 µm to 550 µm. As shown in Figure 2b, dry NdFeB powder was filled into the pre-etched cavities using the doctor blade method and subsequently compacted under low pressure in the cavities. Thereafter, the powder-filled wafers are loaded into a thermal ALD reactor (Picosun R-200). Trimethylaluminum (TMA, STREM Chemicals GmbH, Newburyport, MA, USA) and deionized water are used as precursors for the aluminum oxide layer. Low temperature ALD at 75 °C is performed to minimize the impact of process temperature on the magnetic properties and cracks due to residual stress between the agglomerated structure and the enclosed silicon substrate [16]. Firstly, as depicted in Figure 2c, the powder-filled wafer in the chamber is exposed in the first half-cycle to TMA precursor followed by a nitrogen purge cycle to remove unreacted precursor and byproducts from the chamber. Consecutively, in the second half-cycle, water vapor is pumped into the chamber, forming an aluminum oxide layer (Al_2_O_3_) by reacting with the TMA, and the byproducts were purged away. The thickness of the deposited layer is controlled by the number of sequential precursor cycles. The typical film thickness of Al_2_O_3_ in this study is 75 nm [18]. A comprehensive discussion of the agglomeration process detailing process parameters can be found elsewhere [15]. 

After the agglomeration process, post-processing is carried out to remove particle residues from the front and back of the wafers (Figure 2d). The photoresist layer utilized for the patterning and etching of cavities into the silicon wafers is employed as a sacrificial layer to remove particles in the areas surrounding the micromagnets [18]. O_2_ plasma followed by lift-off in organic solvent is carried out to etch the sacrificial layer. The back of the wafers is conditioned by grinding and polishing, which results in the removal of approximately 20 µm of silicon. For this study, the processed wafers are divided into sets for further investigation. A first set for corrosion stability tests is coated with a 3-µm thick passivation layer of silicon oxide deposited by plasma-enhanced chemical vapor deposition (PECVD) (Figure 2e). A second set is utilized to characterize the typical magnetic properties and thermal stability of the agglomerated NdFeB micromagnets. The aforesaid experiments are investigated at chip level, i.e., performed on individual test structures with dimensions of $\left(2000\times 2000\times 530\right){\mu m}^{3}\;$(L×B×T). For that purpose, the processed wafers are diced into chips. For best comparability, the different chips are picked from the same areas of each wafer.

### 2.2. Characterization Technqiues

The magnetization and demagnetization curves for the micromagnets are measured using a vibrating sample magnetometer (VSM, LakeShore 7400, Carson, CA, USA) by applying fields up to 2 T at room temperature. Scanning electron microscopy (SEM) and focused ion beam (FIB) preparation are used for structural characterization. 

Nanoscale structural analysis of NdFeB5 powders was performed on a Tecnai F30 G² (field emission gun, 300 kV, Thermo Fisher Scientific, Waltham, MA, USA) transmission electron microscope (TEM) equipped with a Si(-Li) X-ray detector (EDAX) for element analysis by energy-dispersive X-ray spectroscopy (EDX). The chemical composition of the metal elements was determined with TEM EDX to be Nd (11.9 ± 0.7) at%, Fe (80.9 ± 1.2) at%, Co (7.2 ± 1.5) at% with less than 10 at% of oxygen.

For the thermal stability study, the micromagnets are heat treated in a laboratory oven under air at temperatures from 75 °C to 425 °C. The temperature is increased in steps of 50 °C, with an annealing time of one hour at each step. After each annealing step, the micromagnets are allowed to cool down and subsequently re-magnetized by applying a field of 2 T at room temperature. Then, the magnetic properties are measured again via VSM. 

TEM in situ heating experiments were conducted using a heating sample holder (Gatan Inc.) and addressing temperatures from 25 °C to 400 °C in steps of 100 °C (heating rate: 20 °C/s, temperature holding time: 30 min). To investigate the mechanism of thermal decomposition, selected-area electron diffraction (SAED) experiments using an aperture with virtual diameter of 250 mm and EDX linescans across the Al_2_O_3_/NdFeB interface were performed at each temperature. Cross-section samples of the NdFeB5 agglomerated powders were extracted from the surface of the PowderMEMS structures and thinned to electron transparency by FIB. The ion-beam milling of NdFeB material resulted in a beneficial redeposition of material, embedding the particle structures in a Fe-rich matrix to enhance the structural integrity of the sample.

Reversible temperature coefficients are calculated by measuring the hysteresis loops in-situ at temperatures varied in the range of 25–425 °C in steps of 25 °C. For the investigation of durability, samples with and without an additional silicon oxide passivation layer are subjected to a pressure cooker test (PCT) for 96 h at 120 °C and 100% relative humidity. Additionally, a different set of samples are stored in DI water for 50 days to assess the corrosion stability of the NdFeB micromagnets. Magnetic properties are measured before and after the PCT and water immersion experiments.

## 3. Results and Discussion

### 3.1. Structural Characterization

**Figure 3:**
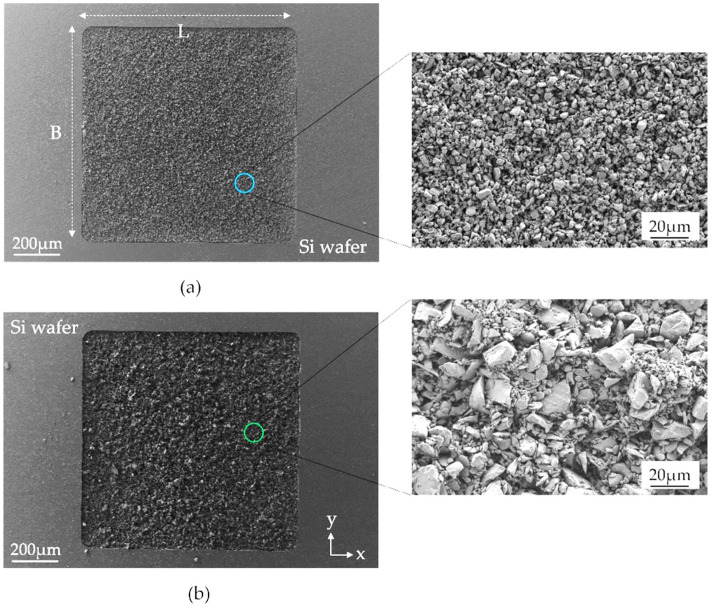
SEM images of top view of the surface of the ALD agglomerated micromagnets. Note that the particles shown in the images are already solidified by an Al_2_O_3_-ALD layer. (**a**) NdFeB5 (d50 = 5 µm) and (**b**) NdFeB25 (d50 = 25 µm). The detailed micrographs on the right side depict the granular structure of the micromagnets.

The PowderMEMS micromagnets fabricated of two different NdFeB particle sizes are shown in Figure 3. The two different particle size distributions are clearly distinguishable in the close-up SEM micrographs, whereas the solidifying Al_2_O_3_-ALD layer cannot be visually separated. 

**Figure 4:**
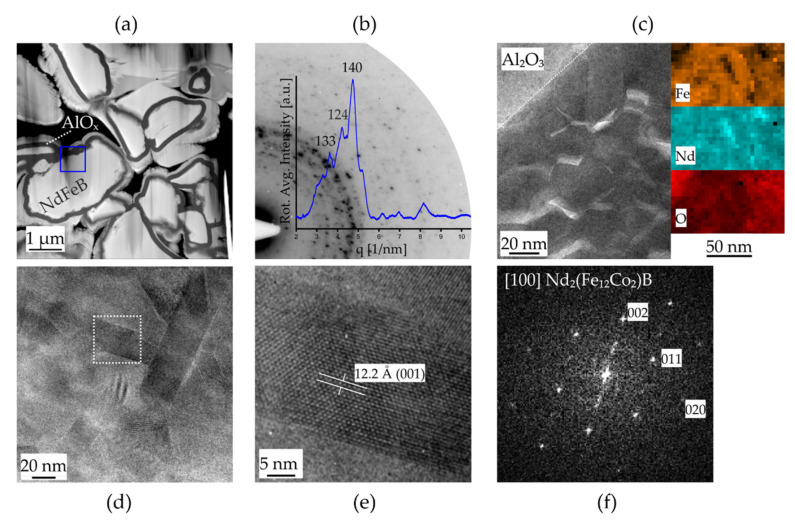
TEM investigation of NdFeB5 [Nd_2_(Fe_12_Co_2_)B] micropowder. (**a**) Scanning TEM image showing the microstructure morphology of NdFeB5 particles coated by a layer of amorphous Al_2_O_3_. (**b**) SAED pattern (inverted contrast) and its rotational intensity distribution of the area marked by the blue frame in (**a**). (**c**) HRTEM micrograph of the Al_2_O_3_/NdFeB interface region and STEM EDS mappings. (**d**) HRTEM micrograph of the nanocrystalline grain structure and (**e**) magnified view of one nanograin and (**f**) FFT image in [100] orientation confirming the tetragonal phase of Nd_2_(Fe_12_Co_2_)B.

Figure 4 shows the results of the TEM investigations on individual micromagnet particles coated with a ~130 nm thick layer of Al_2_O_3_. Cross-sections of the microparticle agglomerates are depicted in the scanning TEM image in Figure 4a. A sufficiently electron transparent region on the sample used for all further investigations is highlighted by the blue frame. SAED experiments on this position of the NdFeB particle yield a speckle pattern of diffracted intensity distributed on rings around the center which is typical for randomly oriented nanocrystalline structures. The first quadrant of this SAED pattern is shown in Figure 4b with its rotationally integrated intensity distribution. The strongest intensity peaks are indexed matching to the reflections of highest intensity of the tetragonal phase of Nd_2_(Fe_12_Co_2_)B [spacegroup: *P*4_2_/*mnm*] [19]. A closer look on the nanocrystalline structure by high-resolution TEM imaging (HRTEM) is given in Figure 4c together with elemental maps recorded on a comparable sample region. Individual grains (dark contrast) are visible close to the interface to the Al_2_O_3_ layer which are occasionally separated by grain boundary (GB) layers (bright contrast). The elemental analysis suggests that phase separation and the formation of Nd- and O-enriched GB oxide layers close to the particle surface occur during the agglomeration process. The presence of metallic Nd GB layers acting as magnetic isolation layers is generally accepted to control the uniquely hard magnetic properties of NdFeB-based alloys. In addition, GB oxide layers containing the crystalline phase Nd_2_O_3_ were recently reported in support of the present observation [20]. The HRTEM analysis is further extended to individual grains, as shown in the images of Figure 4d–f. The individual grains are of tetragonal shape with diameters of 20–30 nm and length 3–4 times their diameter. Figure 4d shows an HRTEM micrograph of the (001) planes and the corresponding reciprocal space image (Figure 4f: Fast Fourier transform) showing the [100] zone axis pattern, which is related to the tetragonal phase of the Nd_2_(Fe_12_Co_2_)B structure.

### 3.2. Magnetic Properties

**Figure 5:**
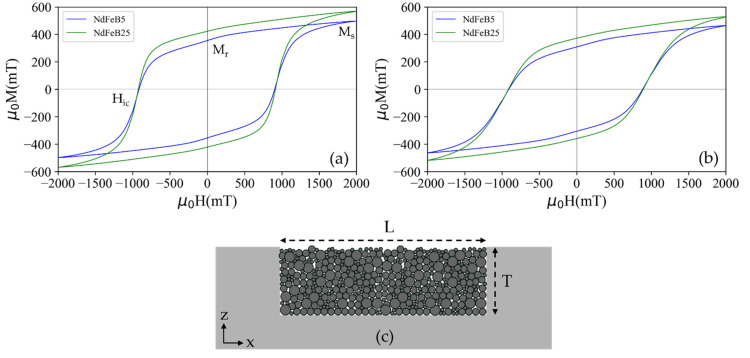
Magnetic curves of the PowderMEMS micromagnets, fabricated using NdFeB powder of particle sizes d50 = 5 µm (NdFeB5) and d50 = 25 µm (NdFeB25). Hysteresis loops of the micromagnets measured in direction of (**a**) x-axis and (**b**) z-axis at room temperature. (**c**) Cross-sectional schematic of the measured micromagnet structure.

The relevant magnetic properties of individual magnetic test structures were investigated by VSM. Figure 5 shows the M-H hysteresis loops of the ALD agglomerated NdFeB micromagnets for the two different powder sizes. The measurements were conducted in the direction parallel to the *x*-axis, i.e., in the substrate plane (Figure 5a), as well as parallel to the *z*-axis, i.e., out of the substrate plane (Figure 5b). Table 1 summarizes the remanence $M_{r}$, intrinsic coercivity $H_{\mathrm{ic}}$ and saturation magnetization $M_{s}$ at maximum applied field (2 T) derived from the hysteresis loops. 

The coercivity measured for the agglomerates is within the range of µ_0_H_ic_ = 880–1050 mT specified by the supplier for the utilized powders [17]. Thus, the particles are individually fixated throughout the entire volume of the solidified structure. The observed remanence magnetization of µ_0_M_r_ = 355–423 mT is lower than the range of µ_0_M_r, bulk_ = 865–895 mT specified by the manufacturer for bulk bonded magnets made by the same powder. This difference is due to the porous structure of the ALD-solidified micromagnets. Considering the measured magnetization, a volume filling factor of 40–50% is estimated. Note that higher filling factors are achieved using an automated filling process [21].

### 3.3. Magnetization Mechanism

**Figure 6:**
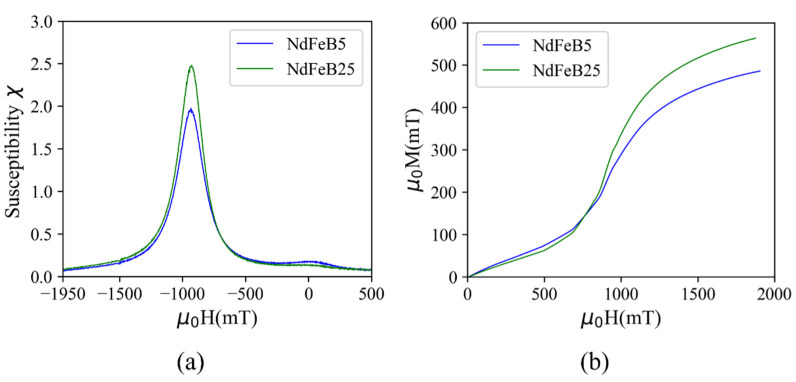
(**a**) Field dependence of the susceptibility χ and (**b**) initial magnetization curves of the ALD agglomerated micromagnets.

The magnetic susceptibility (χ=dM/dH) curves calculated from the M-H curves of Figure 5 are depicted in Figure 6a, through which magnetization reversal changes are identified. Two susceptibility peaks are observed in the case of NdFeB5, namely one pronounced peak at applied fields close to $H_{\mathrm{ic}}$ and a minor one close to remanence. The observation of two peaks indicates the occurrence of magnetization reversal at different applied fields due to different magnetic phases of the particles. The peak at high fields reflects reversal of a hard magnetic volume phase in the material and the additional peak at low fields corresponds to independent reversal of low coercivity materials (soft magnetic phase) at the surface of the particles. The appearance of a minor soft magnetic phase at low applied fields in the case of NdFeB5 micromagnets might be due to oxidation of particles during the ALD process since H_2_O is one of the precursors for the deposition of the Al_2_O_3_ layer. The majority of the byproduct material phases formed after the oxidation reaction in the NdFeB material are soft magnetic in nature [22]. However, the NdFeB powder with larger particle size exhibits a lower surface area to volume ratio, with accordingly slower kinetics of the oxidation reaction. Consequently, the susceptibility peak at lower fields is much less pronounced in the case of NdFeB25, indicating the presence of a nearly pure hard magnetic phase. The presented explanation of the magnetization mechanism will be subjected to future investigation. 

The initial magnetization curves for demagnetized samples from zero to maximum applied field are given in Figure 6b. Both micromagnets demonstrate a two-step magnetization behavior for virgin magnetization. At low applied fields, the magnetization increases gradually, while at fields close to $H_{\mathrm{ic}}$ the magnetization increases rapidly and then approaches saturation, which is not fully achieved at the maximum field of 2 T. The observed magnetization suggests a combination of pinning and nucleation mechanisms, which is commonly recognized in melt-spun NdFeB alloys as used in this study [23]. 

Additionally, the magnetic interactions responsible for magnetization reversal mechanism explained above in ALD agglomerated magnetic material is quantitatively studied by measuring two different field dependent remanence curves, called isothermal remanence $M_{\mathrm{IRM}}\left(H\right)$ and dc-demagnetization remanence $M_{\mathrm{DCD}}\left(H\right)$. Both remanence curves are related by the Wohlfarth equation as [24],

(1) $$m_{d}\left(H\right)=1-2m_{r}\left(H\right)$$

where $m_{d}\left(H\right)=M_{\mathrm{DCD}}\left(H\right)/M_{r}\left(H_{\max}\right)$ is the normalized dc-demagnetization remanence, $m_{r}\left(H\right)=M_{\mathrm{IRM}}\left(H\right)/M_{r}\left(H_{\max}\right)$ is the normalized isothermal remanence and $M_{r}\left(H_{\max}\right)$ is the remanence after applying the maximum applied field of 2 T. The relation is valid for assemblies of interacting and non-interacting, single and multi-domain particles, and thin-film medium [23]. Deviation from relationship (1) indicates that interaction between neighboring grains or particles occur. 

**Figure 7:**
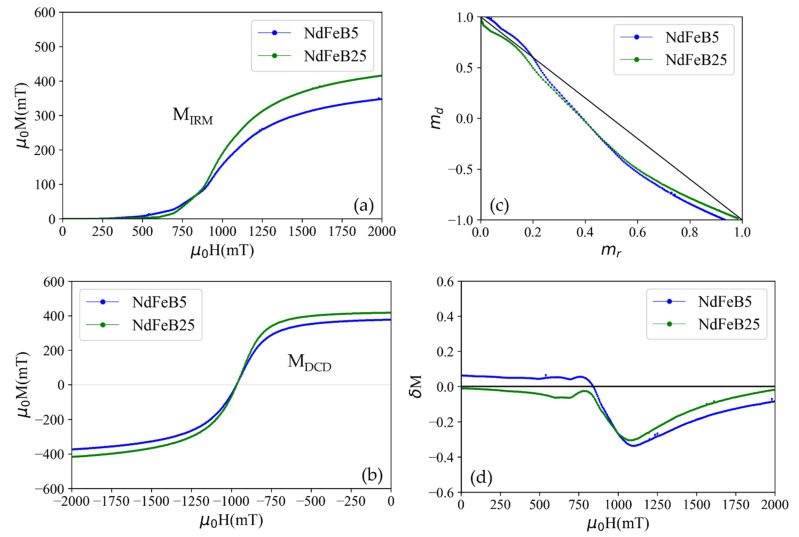
(**a**) Isothermal remanent ($M_{\mathrm{IRM}}$) and (**b**) dc-demagnetization remanent magnetization ($M_{\mathrm{DCD}}$) curves. (**c**) Normalized demagnetization remanence $m_{d}\left(H\right)$ vs. normalized isothermal remanent magnetization $m_{r}\left(H\right)$ for dc demagnetizations of the NdFeB5 and NdFeB25 micromagnets showing long-range magnetostatic interaction. The straight black line represents $m_{d}\;$= 1–2$m_{r}$, corresponding to non-interacting, single domain particles. (**d**) δM plots as a function of external applied magnetic fields.

For measuring the $M_{\mathrm{IRM}}\left(H\right)$ curve, initially, a positive field H was applied to a demagnetized sample. Then the field was removed and the remanence $M_{\mathrm{IRM}}\left(H\right)$ was measured. This process was repeated at incrementally increased fields H until the maximum applied field $H_{\max}$ is reached. An isothermal remanence magnetization curve (Figure 7a) is plotted with the measured remanences as a function of the applied field. 

Similarly, for the measurement of $M_{\mathrm{DCD}}\left(H\right)$, the samples were primarily saturated by applying a maximum field $H_{\max}$ in positive field direction and the remanent magnetization was measured after removing the field. Followingly, the samples were exposed to fields in negative direction with successively increasing field strength up to maximum applied field $-H_{\max}$. The remanent magnetization was measured at each applied negative field value. The measured $M_{\mathrm{DCD}}\left(H\right)$ curve is shown in the Figure 7b. 

The $m_{d}\left(H\right)$ versus $m_{r}\left(H\right)$ curves of the NdFeB5 and NdFeB25 micromagnets are shown in Figure 7c. The black line indicates the Wohlfarth linear relationship corresponding to ideal non-interacting single domain particles. The curves of both micromagnets show deviation towards the region below the line. Such a deviation towards the region below the Wohlfarth line indicates long-range demagnetizing interactions, i.e., interactions that stabilize the demagnetized state [25]. However, for the NdFeB5 micromagnets, the left-hand part of the curve is above the Wohlfarth line which can be attributed to interactions promoting the magnetized state (exchange coupling interactions). Additionally, δM plots measured using Equation (2) are shown in Figure 7d [26].

(2) $$\delta M=m_{d}\left(H\right)-\left(1-2m_{r}\left(H\right)\right)$$

δM plots show the magnetic interaction deviation relationship (2) as a function of external applied magnetic field. δM=0 indicates the non-interacting single domain particles, while positive and negative δM values denotes exchange and long-range interactions respectively [27]. In the case of the NdFeB5 micromagnet, the broad δM peak at low applied fields indicates weak and not well coupled short-range exchange interaction [28]. The presence of such a interaction might be due to the appearance of soft magnetic phase grain in a single phase alloy material [29,30] or interactions between two soft magnetic phases [31]. Additionally, the higher dominant long-range interaction in the case of the NdFeB5 micromagnet at higher applied fields is attributable to an increase in demagnetizing long-range interactions between the NdFeB5 particles due to a low filling factor (Section 3.2) [32]. 

This is in alignment with the observation that the conversion of the shell region of the particles into soft-magnetic species during the oxidation process creates a relevant contribution to the hysteresis behavior (see Figure 5a). Furthermore, the observation in Figure 7d and from the HRTEM micrograph (Figure 4c) suggests that the soft magnetic phase in the particles consists of individual grains that are either in direct contact with each other or are only separated from each other by a hard magnetic phase material. Otherwise, no exchange interaction would take place since the contact area between grains of hard-soft or soft-soft phases has to be large for the continuance of the short-range exchange interaction [33]. 

### 3.4. Thermal Stability

The thermal stability and the influence of elevated temperatures on the magnetic properties are important aspects for the application of PowderMEMS micromagnets in MEMS devices. 

First, experiments were performed to investigate the irreversible degradation of magnetic properties by exposing the micromagnets to a temperature range relevant for back-end-of-line (BEOL) post-processing and for applications, such as automotive and consumer electronics [34,35]. The samples are subjected to heat treatments (75 °C to 425 °C) in air for 1 h in steps of 50 °C as described in Section 2.2. After each annealing step, the samples cool down and are subsequently re-magnetized. Then, $M_{r}$ and $H_{\mathrm{ic}}$ of the micromagnets are measured.

**Figure 8:**
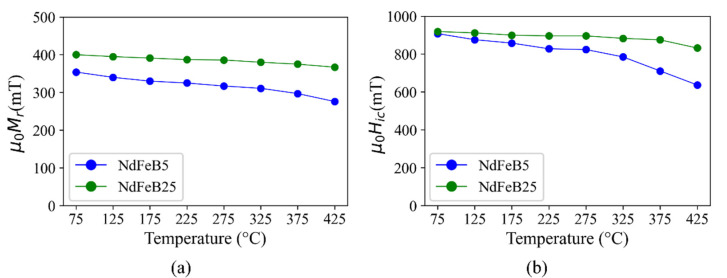
Comparison of irreversible degradation of (**a**) remanence and (**b**) intrinsic coercivity of NdFeB5 and NdFeB25 micromagnets after annealing at different temperatures. For each data point the sample is annealed in air for 1 h, followed by a cool down and re-magnetization at 2 T.

As shown in Figure 8, the degradation of $M_{r}$ and $H_{\mathrm{ic}}$ at 425 °C is for the NdFeB5 micromagnets 22% and 30%, respectively, and in the case of NdFeB25 8% and 9.5%, respectively. When considering the automotive and consumer electronics applications typical operating temperatures are less than 200 °C [34]. The degradation of $M_{r}$ and $H_{\mathrm{ic}}$ at such temperatures is for NdFeB5 7% and 5.5%, respectively, and 2% for both properties in the case of NdFeB25. 

**Figure 9:**
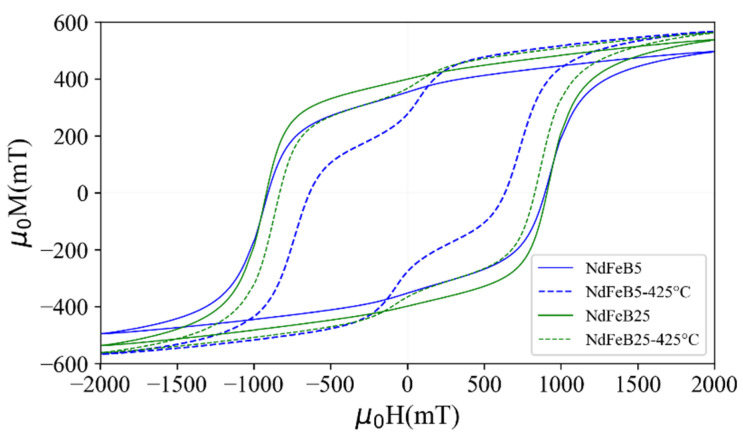
Hysteresis loops of the micromagnets NdFeB5 and NdFeB25 measured before and after annealing at 425 °C at atmospheric conditions for 1 h compared to the as-fabricated hysteresis loops.

To measure the extent of the temperature induced degradation, the micromagnets are subjected to heat treatments under normal atmospheric conditions. After heating the samples at 425 °C for 1 h, a kink appears in the demagnetization loop close to remanence, as shown in Figure 9. Contrary to the decrease in $M_{r}$ and $H_{\mathrm{ic}}$ after heating to 425 °C, $M_{s}$ increases by 14% for NdFeB5 and 5% for NdFeB25. This indicates a higher magnetic moment of soft magnetic material in the shell region of the hard magnetic NdFeB particles. A soft magnetic α-Fe phase is likely formed from the dissociation of the main NdFeB phase during the oxidation reaction at the applied temperature. Such a soft magnetic phase exhibits a saturation magnetization of 2.15 T compared to 1.61 T for Nd_2_Fe_14_B [36,37]. 

**Figure 10:**
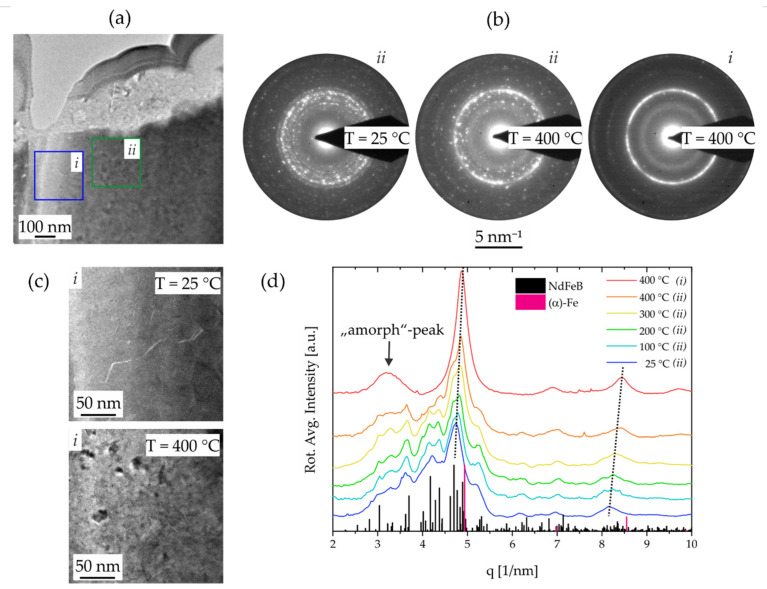
Investigation of the thermal degradation by an in-situ heating TEM experiment. (**a**) TEM image of the Al_2_O_3_-coated NdFeB particle showing two regions of different thickness produced by sample preparation: (*i*) thin region, (*ii*) thick region. (**b**) Exemplary SAED pattern of regions *i* and *ii* recorded at 25 °C and 400 °C. (**c**) TEM images of region I before heating and at 400 °C show the change in nanostructure upon thermal degradation. (**d**) Evolution of radially diffracted intensity (rotational averages) with temperature recorded on region *ii*. Diffracted data is compared to NdFeB and Fe reference data.

The mechanism of the irreversible degradation with temperature was examined in detail by applying defined temperatures to the PowderMEMS micromagnet particles in an in situ TEM experiment. Therefore, the temperature was increased up to 400 °C in steps of 100 °C and SAED experiments as well as EDX measurements were conducted at each temperature to follow possible changes of the nanostructure. The results of this experiment are presented in Figure 10 and indicate the structural degradation of the NdFeB grains into a nanocrystalline Fe-rich phase with cubic α-Fe structure and amorphous species. Figure 10a displays the sample region of the experiments, indicating two areas *i* and *ii* of different sample thickness. Hence, less material is present in case of area *i*. The outcomes of the SAED experiments are summarized in Figure 10b showing the full diffraction patterns before annealing and at T = 400 °C for both areas. An obvious transition from the speckle pattern discussed in Section 3.1 at 25 °C is observed towards a more defined intensity distribution of closed rings in the thin area *i* at 400 °C. The latter pattern is related to a nanocrystalline Fe-rich phase with cubic α-Fe structure. Both patterns are superimposed on the SAED pattern of area *ii* at 400 °C, indicating only partial degradation in larger volumes, e.g., towards the bulk of the particle. The change in the nanostructure is also clearly observed on the TEM images of Figure 10c of the thin area *i* before and after annealing, showing the degradation from large grains into small nanocrystals.

The evolution of the diffracted intensity and hence the structural degradation with temperature is followed by comparing the intensity distribution of SAED patterns recorded at each temperature. The plots of the rotational averaged intensities are shown in Figure 10d for areas *ii* and *i* and are compared with single crystalline reference data of NdFeB (Nd_2_(Fe_12_Co_2_)B [spacegroup: *P*4_2_/*mnm*]) and the α-Fe [spacegroup: *Fm*-3*m*] phase. Here, the first significant changes in the intensity distribution are observed from 300 °C to 400 °C, which is consistent with the reducing magnetic properties in this temperature regime. These changes are q-shifts of the intensity peaks towards the reflection positions of the α-Fe phase with temperature and leveling out of intensity belonging to the parent phase reflections. Afterall, a broad intensity peak at q = 3 1/nm is established, indicating the presence of amorphous phases with only short-range order. EDX line scans across the degraded Al_2_O_3_/NdFeB interface region (not shown) did not show diffusion of elements hence suggesting that the thermal instability is intrinsic.

### 3.5. Corrosion Stability

NdFeB is known to be highly susceptible to corrosion in common ambient environment [38]. Compared to bulk magnets, the effect of corrosion should be higher in the case of the ALD agglomerated micromagnets due to their porous structure. The focus of the following study is to investigate the moisture and oxygen permeation stability of the ALD oxide and passivation layers to determine their stability in certain types of environmental conditions. The experiments are conducted on micromagnets of type NdFeB5 as these should be more sensitive due to their larger surface area. 

**Figure 11:**
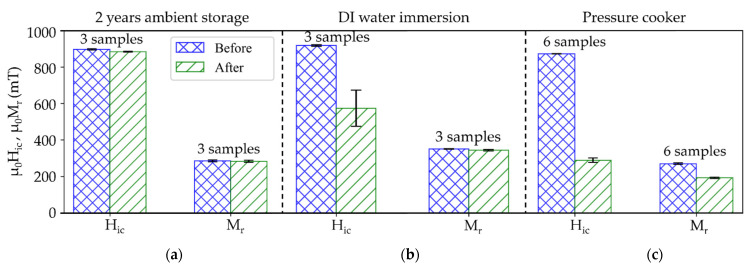
Magnetic properties of unpassivated NdFeB5 micromagnets before and after being subjected to different corrosion stability tests. (**a**) 2 years exposed to ambient conditions, (**b**) immersion test in DI water for 50 days and (**c**) pressure cooker test at 100% relative humidity and 120 °C for 96 h.

A first set of experiments is performed on micromagnets without an additional passivation layer. $H_{\mathrm{ic}}$ and $M_{r}$ are measured before and after exposing the samples to the following three treatments (Figure 11). For each treatment, a pristine set of micromagnets is used. 

First, a long-term ambient air exposure test is conducted by storing the samples in air at controlled indoor conditions (T≤30 ℃, relative humi ≤70%dity) for two years. The micromagnets exhibit virtually no degradation, as shown in Figure 11a. The ALD Al_2_O_3_-layer appears to be sufficient to protect the individual particles from corrosion under such ambient conditions.

**Figure 12:**
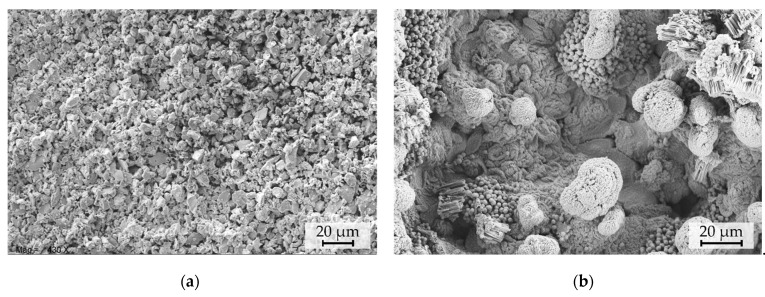
Top-view SEM micrographs of NdFeB5 micromagnets (**a**) after 50-day immersion test and (**b**) 96-h PCT.

Second, an immersion test (IT) is performed by immersing the micromagnets in DI water at room temperature for 50 days. The intrinsic coercivity $H_{\mathrm{ic}}$ decreases by about 48% of its initial value, with a wide spread in observed values. No significant change in $M_{r}$ (Figure 11b) is observed. Since SEM investigations, shown Figure 12a, indicate no visible degradation of the structure, the reason for the decrease in H_ic_ needs to be further investigated.

**Figure 13:**
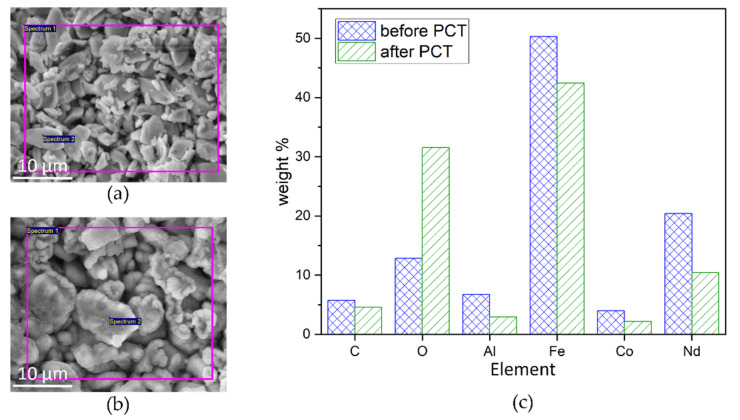
SEM micrographs of NdFeB particles solidified by Al_2_O_3_ (**a**) before PCT and (**b**) after PCT. The violet frames indicate the area of elemental analysis by EDX. (**c**) Results of elemental analysis before and after PCT. A significant increase in O_2_ indicates corrosion of the NdFeB.

Third, a set of micromagnets are subjected to an accelerated heat-humidity stress test (pressure cooker test, PCT) for 96 h at 100% relative humidity and a temperature of 120 ℃. After the PCT (Figure 11c), $H_{\mathrm{ic}}$ is decreased by 60% and $M_{r}$ is 17% lower compared to the initial values. SEM micrographs acquired after PCT treatments, as shown in Figure 12b, depict the degradation of the particulate structure. The morphology appears fissured and exhibits bulging protrusions. These features can be associated with the disintegration of the NdFeB particles and accumulation of Fe-rich oxidation products at the surface due to corrosion promoted by the water vapor under PCT conditions [39]. EDX measurements of the samples before (Figure 13a) and after PCT (Figure 13b) support degradation due to the corrosion of NdFeB since the oxygen content increases significantly (Figure 13c). 

**Figure 14:**
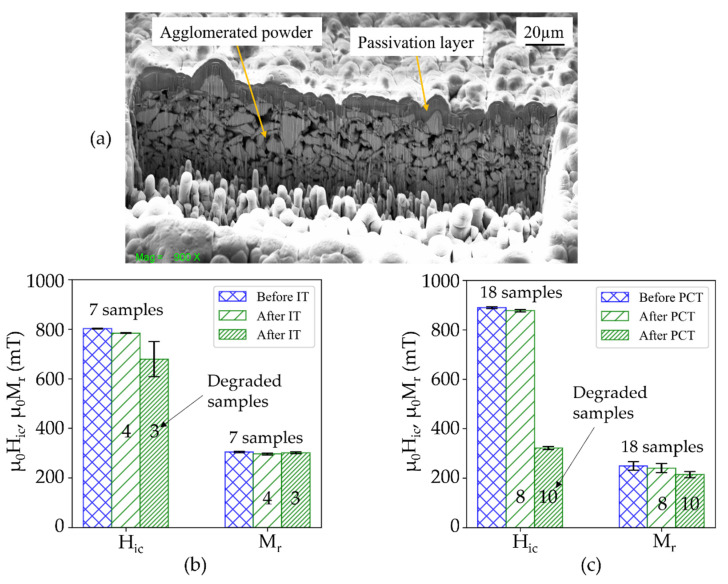
(**a**) FIB-cross section of a passivation layer of 3 µm silicon oxide deposited by PECVD at 400 °C on top of a powder-based NdFeB micromagnet. Magnetic properties of passivated NdFeB micromagnet before and after (**b**) immersion test and (**c**) pressure cooker test. Numbering on top of the error bars indicate measured samples.

Subsequently, NdFeB5 micromagnets equipped with an additional passivation layer of 3 µm silicon oxide were subjected to IT and PCT (sets of 7 and 18 samples, respectively). Figure 14a shows a FIB-SEM cross-section image of a NdFeB5 micromagnet with a silicon oxide passivation layer deposited at 400 °C by PECVD. For approximately half of the samples, the magnetic properties exhibit virtually no degradation, as shown in Figure 14b,c. However, after the immersion test, three out of seven samples, and after PCT 10 out of 18 samples do exhibit degradation. The degradation is most likely due to cracks and pinholes in the passivation layer or the disintegration of the silicon oxide passivation layer. Further work will investigate the failure mechanisms and the suitability of different layers for an effective passivation. Compared to the results without passivation layer (Figure 11), the degradation is significantly less pronounced. This result demonstrates that the degradation of the Al_2_O_3_-agglomerated micromagnets in harsh environments can be effectively inhibited by an additional passivation layer deposited on top of the PowderMEMS structures. 

## 4. Conclusions

High performance voluminous NdFeB permanent micromagnets were fabricated at wafer-level using a MEMS-compatible powder agglomeration process based on low temperature ALD. The magnetic properties of micromagnets fabricated from two NdFeB powders with different particle sizes were investigated. The micromagnets exhibited a remanence $M_{r}$ up to 423 mT and coercive field $H_{\mathrm{ic}}$ up to 924 mT. 

The magnetic susceptibility curve and the Wohlfarth relation indicate that the long-range demagnetizing interaction is the dominant one during magnetization reversal at fields near to the coercive field in both the micromagnets. For micromagnets consisting of smaller particles, the dominance of exchange interaction was observed at lower fields, which could be related to grain boundary oxide layers.

The results of thermal stability tests demonstrate that the PowderMEMS micromagnets are thermally stable within a temperature range relevant for BEOL-processing and MEMS applications. Additionally, passivated micromagnets endured immersion in DI water and the aggressive pressure cooker corrosion test. This stability facilitates the integration of PowderMEMS micromagnets in microfluidic applications and operation in harsh environments.

Future work will advance the endurance of the magnets in harsh environments by developing suitable passivation layers. This includes hermetic layers which prevent the intrusion of gases into the porous structures. Apart from passivation, a focus of future development is on the system-level performance of PowderMEMS micromagnets integrated in various MEMS. Furthermore, PowderMEMS micromagnets made of mixtures of magnetic powders will be used to tailor the effective magnetic properties.

## Figures and Tables

**Table 1:** Magnetic properties of the PowderMEMS NdFeB micromagnets with dimensions of $\left(2000\times 2000\times 530\right){\;\mu m}^{3}$.

	$\mu_{0}M_{r}\left(mT\right)$		$\mu_{0}H_{ic}\left(mT\right)$		$\mu_{0}M_{s}\left(mT\right)$	
	*x*-axis	*z*-axis	*x*-axis	*z*-axis	*x*-axis	*z*-axis
NdFeB5	355	309	915	910	497	464
NdFeB25	423	372	924	915	568	528

## Data Availability

Not applicable.
